# Ancient landscapes and the relationship with microbial nitrification

**DOI:** 10.1038/srep30733

**Published:** 2016-08-02

**Authors:** Sasha N. Jenkins, Daniel V. Murphy, Ian S. Waite, Steven P. Rushton, Anthony G. O’Donnell

**Affiliations:** 1 Soil Biology and Molecular Ecology Group, School of Earth and Environment and the Institute of Agriculture, Faculty of Science, The University of Western Australia, 35 Stirling Highway, Crawley, Western Australia 6009, Australia; 2 School of Biology, Newcastle University, Newcastle upon Tyne, NE1 7RU, England, UK

## Abstract

Ammonia oxidizing archaea (AOA) and bacteria (AOB) drive nitrification and their population dynamics impact directly on the global nitrogen cycle. AOA predominate in the majority of soils but an increasing number of studies have found that nitrification is largely attributed to AOB. The reasons for this remain poorly understood. Here, *amoA* gene abundance was used to study the distribution of AOA and AOB in agricultural soils on different parent materials and in contrasting geologic landscapes across Australia (n = 135 sites). AOA and AOB abundances separated according to the geologic age of the parent rock with AOB higher in the more weathered, semi-arid soils of Western Australia. AOA dominated the younger, higher pH soils of Eastern Australia, independent of any effect of land management and fertilization. This differentiation reflects the age of the underlying parent material and has implications for our understanding of global patterns of nitrification and soil microbial diversity. Western Australian soils are derived from weathered archaean laterite and are acidic and copper deficient. Copper is a co-factor in the oxidation of ammonia by AOA but not AOB. Thus, copper deficiency could explain the unexpectedly low populations of AOA in Western Australian soils.

Nitrification, the aerobic oxidation of ammonia to nitrate, has been central to the global nitrogen cycle since the time when the Earth was first oxygenated^1^. Ammonia-oxidizing bacteria (AOB) and archaea (AOA) carry the ammonia monooxygenase gene, *amoA* that catalyzes the rate-limiting step in nitrification^1^. AOA predominate in the majority of soils but an increasing number of studies have found that nitrification is largely attributed to AOB^2^,^3^,^4^,^5^,^6^,^7^. Much of what we know about soil nitrification comes from work done on agricultural soils of the Northern hemisphere where AOA predominate^8^,^9^,^10^,^11^.

Australian soils have formed on a contrasting geological landscape. The continent is home to some of the oldest Archean rocks (>2500 Ma) in the west, Proterozoic rocks (>541 Ma) in the center of the continent and the most recent Phanerozoic rocks (<541 Ma) in the east. In Western Australia soils are derived from ancient, stable (limited tectonic activity), highly weathered lateritic landscapes^12^,^13^ that over time have given rise to a clay mineralogy dominated by kaolinite^14^. This is particularly true for the agricultural soils in the south-west of Western Australia where the grain growing regions are underlain by the Archaean granitic and gneissic parent rock of the Yilgarn Craton^12^, one of the oldest world land masses. Weathering of this parent rock has resulted in the formation of infertile soils deficient in phosphorus, molybdenum, zinc, copper and other essential elements^13^,^14^,^15^,^16^ that may impose quite different constraints on the evolution of microbial processes such as nitrification and the ecological distribution of ammonia-oxidizing microorganisms^1^. Parent materials are important in pedogenesis and in determining the physiochemical properties influencing microbial biogeochemical cycles in soil^17^,^18^. For example, we know that unlike many soils of the Northern hemisphere, AOB rather than AOA can dominate in some sandy, coarse-textured soils of the Southern hemisphere^2^,^3^,^5^,^6^. The reasons for this are not yet understood but high rates of nitrogen fertilization^3^,^4^ have been implicated. However, a consequence of extensive weathering and subsequent formation of laterite (a thick hard crust of iron oxides)^13^ is that such soils may also be higher in available iron and have a higher redox potential^19^.

To investigate the reasons why AOB, rather than the expected AOA abundances, were higher in the soils of Western Australia^2^,^6^ we investigated the relationships between bacterial and archaeal *amoA* gene abundance and soil chemistry drivers in the primary grain growing regions across Australia (n = 135 sites; Table 1, Figs 1 and 2). These soils spanned a range of geological ages from 1 to 2,800 Ma and included semi-arid, temperate and subtropical agro-ecological zones.

## Results and Discussion

**Figure 1:**
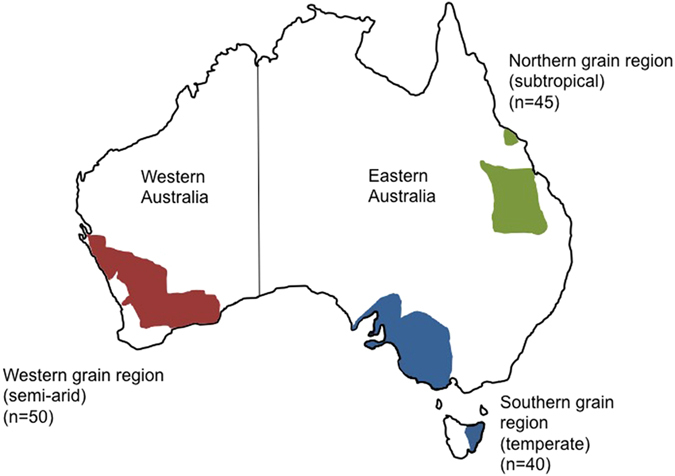
Agricultural soils across Australia. Map shows soil samples (n = 135 sites) were collected from the primary grain growing regions across Australia (Western, Southern and Northern) and agro-ecological zones (semi-arid, temperate, subtropical). The map was drawn using PowerPoint (Microsoft Office 10) using the outline of Australia from https://en.wikipedia.org/wiki/States_and_territories_of_Australia#/media/File:Australia_location_map_recolored.png.

**Figure 2:**
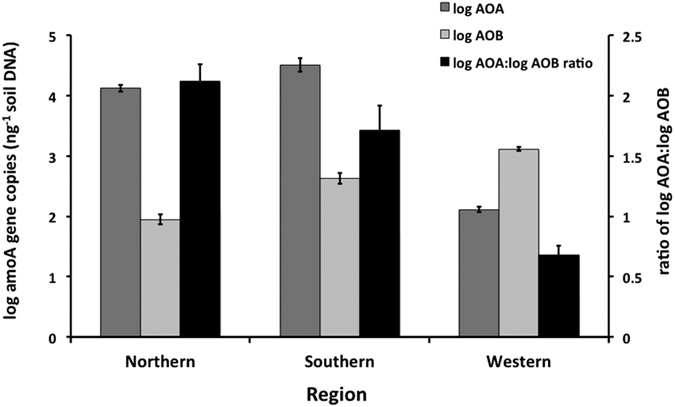
Ammonia oxidizer populations in agricultural soils across Australia. Mean log abundance of ammonia oxidising bacteria, archaea and archaea:bacteria ratio (*amoA* gene copy number) for Western (n = 50), Southern (n = 40) and Northern (n = 45) grain growing regions across Australia.

Our results show that there is a clear shift in the relative dominance of AOB and AOA across Australia with AOB more abundant than AOA in the highly weathered, ancient and acidic soils of Western Australian (mean pH = 4.6 and clay content = 4.9%; Table 1; Figs 1 and 2). In contrast, AOA numbers increased in the younger (derived from Phanerozoic rocks <541 Ma), more fertile soils of the northern and southern grain regions (subtropical and temperate, respectively) of Eastern Australia (mean pH = 6.3 and mean clay content = 37.6%; Table 1, Fig. 2). These findings contradict the primarily Northern hemisphere data where AOA tend to dominate in low pH soils^7^,^9^,^10^,^11^. It has been reported that the high abundance of AOB in these Western Australian soils could be due to the fact that they receive high rates of inorganic N^2^,^3^. However, this can be ruled out here since the Western Australian soils received similar inputs of ammonium-based fertilizers to those used in Eastern Australia (Table 1). This shows that factors other than inorganic nitrogen inputs are responsible for the niche separation of AOB and AOA in these soils.

**Figure 3:**
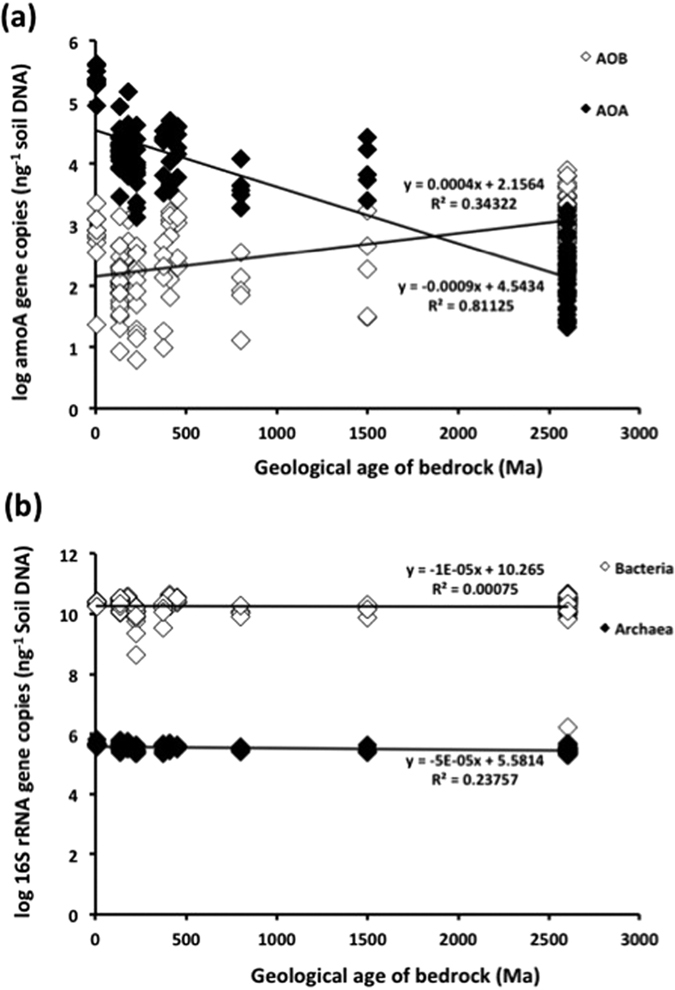
Relationship between geological age in soils and ammonia oxidizer populations in agricultural soils across Australia. The scatter plots show the relationship between geological age in Australian soils and (**a**) log abundance (*amoA* gene copy number) of ammonia oxidizing bacteria (AOB) and archaea (AOA) and (**b**) log abundance (16S rRNA gene copy number) of total bacteria and archaea.

To investigate further, we explored the relationship between the geological age of the underlying parent rock, levels of copper and other soil characteristics and the relative distributions of AOA and AOB. These studies revealed a clear negative correlation between soil AOA populations and the geological age of the underlying parent material (Fig. 3a). Thus, AOA tend to have a strong preference for younger soils. This could explain their widespread distribution and prevalence in Eastern Australia, Europe, North America and China^8^,^9^,^10^ where the soils are geologically younger. This large-scale, geographic association and distribution warrants further investigation. Interestingly, both the total archaeal and bacterial population did not vary with geologic age (Fig. 3b), suggesting that the chemical or physical status of the resulting soils may impose other constraints on ammonia oxidation. The underlying soil parent material and time have been shown to strongly influence the physical and chemical properties of the surface soils^17^, especially nutrient cycling. Ultimately this will influence the microbial populations and the niches they will occupy. This might explain why the ammonia oxidizing microorganisms that mediate nitrification are affected by geological age whilst the overall archaeal and bacterial community size remains unchanged.

**Figure 4:**
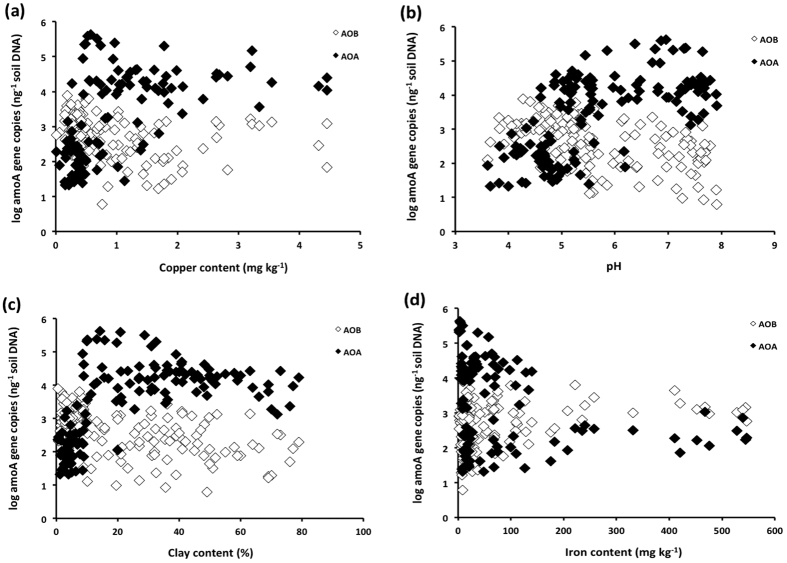
Relationship between soil properties and ammonia oxidizer populations in agricultural soils across Australia. The scatter plots show the relationship between log abundance (*amoA* gene copy number) of ammonia oxidizing bacteria (AOB) and archaea (AOA) and total available copper (**a**), soil pH (**b**), clay content (**c**) and iron (**d**).

Additional analysis of the distribution of AOA and AOB abundances in these Australian soils using generalised linear modelling and structural equation modelling showed that ammonia oxidizer populations were also shaped by physiochemical properties (copper content, clay content, pH) which are largely determined by the soil parent material^17^. The log-transformed abundance of AOA was significantly related to the levels of total available copper and soil pH (Table 2, Fig. 4a,b) whereas the log-transformed abundance of AOB was significantly related only to clay content (Table 2, Fig. 3c). Further analyses showed that the log-transformed ratio of AOA:AOB was inversely related to clay content, copper concentration and pH. It is likely that clay content influences other soil properties (e.g. nutrient availability, soil pH) and through these interactions impacts both directly and indirectly on ammonia oxidizer communities^20^,^21^,^22^. For example, the increased binding of ammonium in clayey soils may impede the growth of ammonia oxidizer populations by reducing the availability of ammonium^22^. Importantly, land use and management practices (e.g. liming, fertilization, tillage) were shown to have had no significant effect on either AOA or AOB abundance.

These findings illustrate that the AOA component of the ammonia oxidizing microbial community was lower in the copper deficient soils of Western Australia (Fig. 3a). AOB abundance was also moderately correlated with the soil iron content (Fig. 3d), with both iron content and AOB abundance greater in Western Australian soils (Table 1). These are interesting functional relationships since recent genomic studies have shown that the metabolism of AOA is fundamentally different from that of their bacterial counterparts and involves a highly copper-dependent system for ammonia oxidation^1^,^23^,^24^; iron on the other hand is a key co-factor in AOB oxidation^1^. These differences in the physiological requirement for copper and iron help explain why AOB rather than AOA predominate in Western Australian soils. We hypothesize that over an extended geological timescale, weathering and erosion of ancient parent rock^12^,^13^,^17^ has selected for microorganisms able to tolerate the resultant iron-rich, nutrient poor soils.

Soil pH is also recognized as a key driver of ammonia oxidizing community structure and function at different agro-ecological scales with most of these studies reporting that at low soil pH (which limits the release of ammonia from ammonium) AOA predominate^7^,^8^ because of their high affinity for ammonia compared to AOB^25^. While AOB are generally not well adapted to low pH environments, some species of AOB and AOA^26^ can overcome the carbon and energy constraints by using alternative metabolic pathways that produce ureases to catalyse the conversion of urea to carbon dioxide and ammonia. Previous studies have also reported on an important role for AOB populations in ammonium oxidation in acidic, semi-arid soils in Chile^5^ and Western Australia^2^,^6^ but did not link this to the geological age of the soils or to the availability of co-factors such as iron and copper.

**Figure 5:**
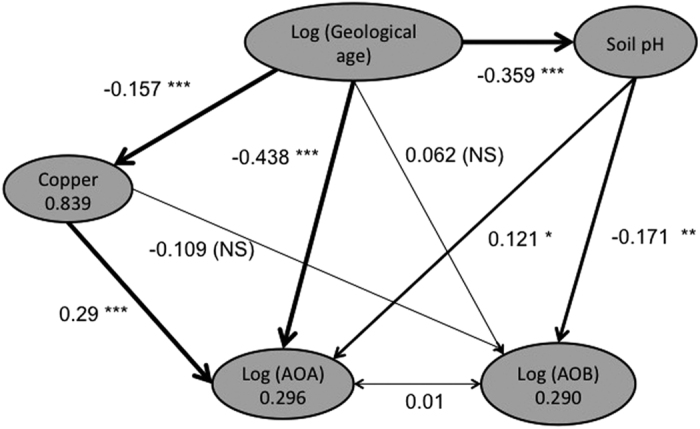
Structural equation model (SEM) investigating drivers of Ammonia oxidizer populations in agricultural soils across Australia. Structural equation models showing impact of log geological age on pH, copper content, and log abundance (*amoA* gene copy number) of ammonia oxidizing bacteria (AOB) and archaea (AOA). Pathway coefficients indicate the impact of a unit standard deviation change at one node on the units of standard deviation change in connected nodes. Thus a unit change in copper content (0.438) has a greater correlation with AOA abundance than pH (0.121). The parsimonious model explained 0.059 of root mean square error of approximation (RMSEA) at 90% confidence interval (0.000–0.270) with a goodness of fit for the model explained 0.999 of the variation in the data. Wald test that coefficient is different to zero (*P < 0.05, **P < 0.01, ***P < 0.001).

Based on the data presented here we hypothesise that the geological age of the parent material and associated soil formation processes have a profound effect on AOA abundance through differences in pH and the availability of copper as a key co-factor in archaeal ammonia oxidation^1^,^23^,^24^. From our data we developed a hypothetical model (*a priori* model) describing the links and interaction between log geological age on pH, copper (Cu) content, and log abundance (*amoA* gene copy number) of AOB and AOA. Structured Equation Modeling (SEM) was used to investigate the relationships within the hypothetical model (Fig. 5). Log AOA was negatively correlated to log geological age and positively correlated to copper content and pH whereas log AOB was negatively correlated to pH only. Log geological age was also negatively correlated to soil pH and copper content. SEM supports our hypothesis that the weathering of ancient Western Australia landscapes affects the availability of these cofactors for AOA oxidation (copper) and AOB oxidation (iron) and that AOA and AOB exhibit different ecophysiologies and occupy different ecological niches in soil^2^,^7^. These results provide further evidence of the importance of bacteria in ammonia oxidation in the topsoil of semi-arid agricultural soils^2^,^6^,^27^.

## Methods

Soil samples (0–10 cm) were collected from 135 monitoring sites (Fig. 1) spanning the major agricultural zones of Australia (described in Sanderman^28^). This includes the summer rainfall subtropical regions of New South Wales and Queensland; the Mediterranean-type, winter dominated rainfall zones of Western Australia; the moist southeastern soils of South Australia; the dry marginal southeastern regions of Victoria and the high rainfall eastern zones of Tasmania. Within each monitoring site (20 m^2^) soil was collected from 10 random locations to form a *ca.* 2 kg composite sample. Soil properties, climate data and management histories (previous 5–10 years) were recorded for each site^29^ as summarized in Table 1. Soils were collected field dry, during summer fallow outside of the period of active plant growth when microbial populations are known to be more stable. Soils were classified as mostly Tenosol or Sodosol in Western Australia, Chromosol in South Australia, Sodosol and Calcarosol in Victoria, Vertosol in Queensland and Ferrosol and Dermosol in Tasmania using the Australian Soil classification series^30^. Soil texture was determined by particle size analysis and characterized according to the Australian Field Texture Grade adapted from McDonald *et al.*^31^ and ranged from predominantly sandy soils in Western Australia, loamy and light clay soils in Victoria and South Australia and medium to heavier clays in Tasmania and Queensland.

The geologic age of the underlying parent rock was determined by plotting the sample coordinates on the relevant 1:250,000 geological maps (http://www.geoscience.gov.au) for each agro-ecological region. Soils across Australia are derived from parent rock of varying age including granite dating back to the Archaean period in Western Australian (~2800 Ma); granite from the Proterozoic period (635–2500 Ma) in South Australian; Tasmanian granite and dolerite from the Devonian (<423 Ma) and Jurassic (<208 Ma), respectively; siltstone and sandstone in Queensland from the Devonian (<375 Ma) through to the Jurassic (<180 Ma) and basalts and sandstones from the tertiary (<31 Ma) and Quaternary (0.7 to 3.6 Ma) period in Victoria, Queensland and Tasmania.

Land management was classified as pasture (n = 30), continuous cropping (n = 40) or mixed cropping (n = 65) where cropping is in rotation with annual pastures. Pasture management was further divided into annual (i.e. no growth over summer fallow) or perennial pastures that were either grass or legume dominated or a mixed grass/legume sward. Grazing management options were no grazing, set stocked or rotational grazing. Tillage practices were classified as zero till (no soil disturbance other than sowing), minimum tillage (one soil working in addition to sowing) or conventional tillage (two or more soil workings in addition to sowing). Stubble management options included: all stubble retained on soil surface, all stubble retained and worked into soil, stubble grazed, stubble baled and removed, or stubble burnt. Lime (n = 40) or gypsum (n = 5) had been applied to some soils.

The collected bulk soils were sieved (<4 mm) prior to soil characterisation and analysis. Bulk density, water holding capacity, cation exchange capacity (CEC), electrical conductivity (EC), pH and particle size analysis were determined for each soil as described previously^32^,^33^,^34^. The EC and pH were measured (1:5 soil:water ratio) using a probe inserted into water or 0.01 M CaCl_2_ mixtures, respectively. Calcium, magnesium, aluminium, silica, copper and iron content of each soil were measured by inductively coupled plasma emission spectroscopy (Perkin Elmer Optima 5300DV ICP-OES). Total phosphorus and potassium was measured using standard methods^32^. Total carbon and nitrogen were measured by combustion using an Elementar Analyser (Vario Macro CNS; Elementar, Germany).

DNA was extracted from the soils as described previously^35^, quantified and checked for purity at A260/280 nm (Nanodrop, Thermo Fisher Scientific, USA), before adjusting to 4 ng μl^−1^ using molecular grade water (Sigma-Aldrich, Sydney, Australia) and stored at −20 °C. The abundance of the AOA and AOB were quantified by quantitative polymerase chain reaction (qPCR) using an Applied Biosystems 7500FAST qPCR instrument (Life Technologies, Victoria, Australia) using oligonucleotide primers specific for archaea and bacteria^2^,^27^.

Amplification was carried out in a 20 μl reaction containing, 10  μg  μl^−1^ of BSA (Ambion, Life Technologies, Victoria, Australia), 20 ng of template DNA, 200 nM of primers, 7.8 μl of sterile molecular grade water and 10 μl of GoTaq qPCR mastermix (Promega, Madision WI USA). The cycling conditions for each primer set have been previously described^2^,^27^. All amplifications were performed in triplicate and included a set of DNA standards over four orders of magnitude that were used to construct a standard curve. Molecular grade water (no template) was used as a negative control throughout. Standard curves were generated for each primer set based on triplicate tenfold dilution series of a known amount of linearized cloned plasmid DNA (pCR4-TOPO, Invitrogen, Life Technologies, Victoria, Australia) as described previously^2^,^27^.

The PCR results were accepted if the linear regression of the standard curve had an R^2^ > 0.98 and amplification efficiencies of between 80% and 110%. Variation among replicates was determined by the coefficient of variation (CV) and ranged from 0.11 to 3.14. After amplification, a melting curve was acquired to verify specificity of the qPCR products. Fluorescence data was only collected at temperatures above the Tm of the primers but below that of the target (78 °C for both amoA genes, 72 °C for archaeal and 75 °C for bacterial 16S rRNA genes)^4^.

The amoA gene copies for AOA, AOB and AOA:AOB ratio were log transformed prior to data analysis. The relationships between abundance of AOA and AOB and the measured environmental variables reported in Table 1 were analyzed using generalized linear modeling (GLM). Residuals were assessed for normality using quantile plots and all regression analyses were performed using the R statistical package^36^.

Structural Equation Modelling (SEM) is an *a priori* modelling approach used to test hypothesised pathways of causal links, and quantify them in terms of the unit effect of each node in the path on the next, and then identify the most parsimonious model that explains the observed data^37^. SEM was used to investigate the impact of log geological age on soil pH, copper content and AOA and AOB abundance in the priori model. The model tested for each factor (variables) and possible relationship with AOA or AOB, whilst non-significant pathways were removed. The goodness of fit for the model to explain variation in the data due to relationships between variables was done as described previously^37^. All SEM modeling analyses was performed using the lavaan library in the R statistical package^36^.

## Additional Information

**How to cite this article**: Jenkins, S. N. *et al.* Ancient landscapes and the relationship with microbial nitrification. *Sci. Rep.* **6**, 30733; doi: 10.1038/srep30733 (2016).

## Figures and Tables

**Table 1:** Soil properties, climate data and management history for samples (n = 135) that span the agricultural zones across Western and Eastern Australia*.

Climate, soil and management description	Eastern Australia				Western Australia			
	Mean	SE	Min	Max	Mean	SE	Min	Max
Archaea (log amoA gene copies ng^−1^ soil DNA)	4.3	0.1	3.1	5.6	2.1	0.0	1.3	3.2
Bacteria (log amoA gene copies ng^−1^ soil DNA)	2.3	0.1	0.8	3.4	3.1	0.0	2.1	3.9
Archaea (log 16S rRNA gene copies ng^−1^ soil DNA)	10.3	0.0	8.7	10.7	10.3	0.1	6.2	10.7
Bacteria (log 16S rRNA gene copies ng^−1^ soil DNA)	5.6	0.0	5.3	5.8	5.5	0.0	5.3	5.7
Latitude (dec °)	−32	1	−43	−23	−35	0	−35	−34
Longitude (dec°)	147	0	136	151	118	0	118	118
Annual rainfall (mm)	611	20	403	1348	553	7	422	680
Annual temperature (°C)	17	0	10	23	16	1	15	16
Geological age of bedrock (Ma)	256	40	1	1852	2800	0	2800	2800
Clay (%)	38	2	7	79	5	0	1	20
Silt (%)	14	1	2	26	1	0	0	4
Sand (%)	49	2	4	89	94	0	78	99
Bulk Density (g cm^−3^)	1.1	0.0	0.7	1.5	1.4	0.0	1.2	1.6
pH (CaCl_2_)	6.3	0.1	4.6	7.9	4.7	0.0	3.6	6.2
Dissolved organic carbon (mg C kg^−1^)	130	20	13	901	120	4	39	289
Carbon (%)	2.2	0.2	0.5	10.1	2.8	0.1	0.7	9.8
Nitrogen (%)	0.2	0.0	0.0	0.8	0.2	0.0	0.0	0.7
Phosphorus (mg kg^−1^)	46	4	4	181	29	2	6	97
Calcium (meq 100 g^−1^)	13	1	1	50	4	0	2	11
Potassium (meq 100 g^−1^)	1	0	0	2	0	0	0	1
Magnesium (meq 100 g^−1^)	4	0	0	19	1	0	0	2
Copper (mg kg^−1^)	1.0	0.1	0.3	4.5	0.5	0.1	0	1.7
Iron (mg kg^−1^)	35	4	2	140	152	16	7	547
Electrical conductivity (dS m^−1^)	1	0	0	3	2	0	0	5
Cation Exchange Capacity (meq 100 g^−1^)	19	1	2	58	6	0	2	14
Water holding capacity (%)	55	2	25	102	42	1	28	73
N Fertilizer (Kg N ha^−1^)^a^	34	2	8	64	33	3	1	180

^*^Eastern Australia soils include the northern and southern grain growing regions while western soils cover the Western Australian grain growing region. Values represent the mean, standard error (SE), maximum (Max) and minimum (Min).

^a^Fertilizer application is based on a cereal or oilseed crop.

**Table 2:** Generalised linear models (GLMs) exploring the relationship between log AOA, log AOB and the ratio of log AOA:AOB and variables clay content, rainfall, pH and copper*.

Model	Variable	Estimate	SE	T value	P value
log(AOA)	pH	1.4221	0.1670	8.513	1.22e–13***
	Copper	1.2021	0.1952	6.158	1.34e–08***
log(AOB)	Clay	−0.03753	0.00563	−6.666	6.41e–10***
log(AOA:AOB ratio)	Clay	−0.0536	0.01665	−3.219	0.00171**
	pH	−1.48538	0.25203	−5.894	4.62e–08***
	Copper	−0.91078	0.30293	−3.007	0.00331**

*These parsimonious models explained (54.6%, 25%, and 62.1%, respectively) of the total variance in the data and had an intercept of 1.393, 6.832 and 8.887, respectively. Values represent the estimate, standard error (SE), T value and P value. Relationships shown to be significant are reported at the 0.001 to 0.05 levels (*P < 0.05, **P < 0.01, ***P < 0.001).
